# Prehospital intubation of the moderately injured patient: a cause of morbidity? A matched-pairs analysis of 1,200 patients from the DGU Trauma Registry

**DOI:** 10.1186/cc10442

**Published:** 2011-09-13

**Authors:** Bjoern Hussmann, Rolf Lefering, Christian Waydhas, Steffen Ruchholtz, Arasch Wafaisade, Max Daniel Kauther, Sven Lendemans

**Affiliations:** 1 Trauma Surgery Department, University Hospital Essen, Hufelandstraße 55, 45122 Essen, Germany; 2 Institute for Research in Operative Medicine (IFOM), Faculty of Medicine, Witten/Herdecke University GmbH, Cologne Merheim Medical Center, Ostmerheimer Straße 200, 51109 Cologne, Germany; 3 Trauma Department, Hand and Reconstructive Surgery Unit, University Hospital Marburg, Baldingerstraße, 35043 Marburg, Germany

## Abstract

**Introduction:**

Hypoxia and hypoxemia can lead to an unfavorable outcome after severe trauma, by both direct and delayed mechanisms. Prehospital intubation is meant to ensure pulmonary gas exchange. Limited evidence exists regarding indications for intubation after trauma. The aim of this study was to analyze prehospital intubation as an independent risk factor for the posttraumatic course of moderately injured patients. Therefore, only patients who, in retrospect, would not have required intubation were included in the matched-pairs analysis to evaluate the risks related to intubation.

**Methods:**

The data of 42,248 patients taken from the trauma registry of the German Association for Trauma Surgery (Deutsche Gesellschaft für Unfallchirurgie (DGU)) were analyzed. Patients who met the following criteria were included: primary admission to a hospital; Glasgow Coma Scale (GCS) of 13 to 15; age 16 years or older; maximum injury severity per body region (AIS) ≤ 3; no administration of packed red blood cell units in the emergency trauma room; admission between 2005 and 2008; and documented data regarding intubation. The intubated patients were then matched with not-intubated patients.

**Results:**

The study population included 600 matched pairs that met the inclusion criteria. The results indicated that prehospital intubation was associated with a prolonged rescue time (not intubated, 64.8 minutes; intubated, 82.3 minutes; *P *≤ 0.001) and a higher volume replacement (not intubated, 911.3 ml; intubated, 1,573.8 ml; *P *≤ 0.001). In the intubated patients, coagulation parameters, such as the prothrombin time ratio (PT) and platelet count, declined, as did the hemoglobin value (PT not intubated: 92.3%; intubated, 85.7%; *P *≤ 0.001; hemoglobin not intubated, 13.4 mg/dl; intubated, 12.2 mg/dl; *P *≤ 0.001). Intubation at the scene resulted in an elevated sepsis rate (not intubated, 1.5%; intubated, 3.7%; *P *≤ 0.02) and an elevated prevalence of multiorgan failure (MOF) and organ failure (OF) (OF not intubated, 9.1%; intubated, 23.4%; *P *≤ 0.001).

**Conclusions:**

Prehospital intubation in trauma patients is associated with a number of risks and should be critically weighed, except in cases with clear indicators, such as posttraumatic apnea.

## Introduction

Prehospital intubation of patients after severe trauma is a standard treatment in initial emergency care. According to the current guidelines (for example, the S3 guidelines of the German Association for Trauma Surgery [1], the guidelines of the Eastern Association for the Surgery of Trauma [EAST] [2], and the guidelines from training concepts, such as Advanced Trauma Life Support (ATLS)) [3], four posttraumatic impairments, depending on the respiratory tract, present clear indications for prehospital intubation: (a) posttraumatic apnea or hypoventilation (SaO_2 _< 90%) because the ATLS stipulation "treat first what kills first" applies, making this the highest priority [3]; (b) severe brain injury, usually defined by a Glasgow Coma Scale (GCS) score ≤ 9, in which hypotension is usually the main cause of death apart from hypoxia [4]; (c) hemorrhagic shock with systolic blood pressure ≤ 90 mm Hg; and (d) severe thoracic trauma with respiratory failure (respiratory rate ≥ 29) [2]. However, out-of-hospital airway-management decisions are often difficult, especially in borderline cases. Thus, Ruchholtz *et al*. [5] showed in a retrospective analysis that patients with severe thoracic trauma, but without respiratory failure and prehospital intubation, did not have increased organ failure or mortality. The same result applies to patients with a GCS score ≥ 9. In German-speaking countries, the term *Schutzintubation *(protective intubation) is often used in this context. This term usually refers to protecting the patient against possible aspiration when the patient is assumed not to have an empty stomach.

In addition, indications for prehospital intubation have different evaluations in the current literature. Some rather controversial indications for intubation are pain treatment and immobilization and the sedation of patients displaying psychiatric problems after trauma. In a recent study, Muakkassa *et al*. [6] showed that patients who have been intubated after trauma because of combativeness rather than for a medical indication have identical mortality outcomes but stay longer in the hospital and have more complications, such as pneumonia.

The decision for prehospital intubation is generally associated with a number of possible complications. In a systematic review, Mort *et al*. [7] showed that both hypotension and hypertonia can appear after anesthetic induction. The latter can, for example, intensify potential bleeding. Another possible consequence of intubation is the direct blocking of the respiratory system. Thus, the incidence of malposition of the tube is higher in out-of-hospital intubations. These malpositions involve the esophagus (2% to 9%) or the right mainstem bronchus (15%) [8,9]. Aspiration, both during the induction and afterward, is a risk in patients with full stomachs [6,10]. These complications are eventually associated with a higher incidence of hypoxia and can increase mortality.

After examining the current literature, the following question arises: is prehospital intubation of a moderately injured patient after trauma an independent risk factor? We addressed this question in a cohort of trauma (Abbreviated Injury Scale (AIS) ≤ 3) patients selected from the DGU Trauma Registry who had been intubated before hospitalization but who had no clear indication for intubation based on their prehospitalization status.

## Materials and methods

The trauma registry of the German Society for Trauma Surgery was started in 1993. It contains prospectively collected data from 166 collaborating European trauma centers. The data were entered by hand from patient records until 2001, at which point, data input for central submission was automated via online data-entry software (since 2002). About 100 data elements are collected per patient, including the coding of each injury according to the 1998 revised version of the AIS. The data are submitted to a central database that is hosted by the Institute for Research in Operative Medicine at the University of Witten/Herdecke, Cologne, Germany. Irreversible data anonymity is guaranteed for the patient and participating hospital. Only patients from Germany and Austria were included in this study to minimize variation due to different rescue systems. All of the patients received care from a physician before hospitalization. Records collected between 1993 and 2007 (42,248 total patients) were considered for this study. With the data from the Trauma Registry of the DGU, we received full approval from the ethics committee of the University of Witten/Herdecke, Cologne, Germany.

Patients were selected for this study according to the following criteria:

direct admission from the scene of the trauma; age older than 16 years; GCS, 13 to 15; maximum injury severity per body region (AIS) ≤ 3; no administration of packed red blood cell units in the emergency trauma room; date of admission from 2005 to 2008; and documented data on the intubation.

The patients were divided into a "not intubated" and an "intubated" group, depending on whether they were intubated before hospitalization. To isolate the effect of prehospital intubation, the patients in the two groups were matched according to the following criteria.

1. Abbreviated Injury Scale ≤ 3, with further subdivision into four groups: (a) AIS head ≤ 3, (b) AIS thorax ≤ 3, (c) AIS abdomen ≤ 3, and (d) AIS extremities with pelvis ≤ 3. In individual cases, the triple occurrence of an AIS of 3 was possible for the calculation of the total ISS from the individual body regions.

2. Subdivision into four age groups: (a) 16 to 25, (b) 26 to 55, (c) 56 to 70, and (d) older than 70 years.

3. Male versus female.

4. High-speed trauma versus no high-speed trauma.

Sepsis was defined according to the criteria of Bone, which are close to those of the American College of Chest Physicians/Society of Critical Care Medicine (ACCP-SCCM) consensus conference definition [11]. Single-organ failure was defined as a value ≥ 3, by using the definition determined by the Sequential Organ Failure Assessment (SOFA) score [12]. This value was entered as the total point value by the individual hospitals. Multiple organ failure (MOF) was listed if simultaneous organ failure was recorded in at least two organs. Prehospitalization parameters, hospital stay, and outcome were examined separately for each group.

### Statistics

The data were analyzed with the Statistical Package for the Social Sciences (SPSS; version 17, Chicago, IL, USA). Incidences were represented as the number of cases and percentages and measured by mean values and standard deviations. Differences between the two groups were compared by using the χ^2 ^test for categoric variables and the *t *test for continuous variables. In cases of obvious deviation from normality, continuous variables were tested with a nonparametric rank test (Mann-Whitney). We applied a significance level (α) of 5% to all statistical tests.

## Results

Six hundred injured patients from the DGU Trauma Registry with prehospital intubations were matched with 600 patients without prehospital intubations. The mean age for all the patients was 38.8 years (± 17.1), and 79% were men. The Injury Severity Score (ISS) was not significantly different between the groups (not intubated, 15.1 ± 4.9; intubated, 15.1 ± 5.0). The majority of the injuries were caused by blunt trauma (94.7%). As expected from the matching criteria, the distribution of the injuries by body region was identical between the groups, except for the incidence of facial injuries with an AIS of 1 to 3 (Table 1). There were 3% more patients with such injuries in the prehospital-intubation group than in the group without intubation. However, this difference was not significant (*P *= 0.73). The prehospital respiratory rate was similar in both groups (intubated, 16.5; not intubated, 16.6). As shown in Table 1 heart rate and blood pressure at the trauma scene differed significantly, but clinically relevant differences could not be shown. The survival prognosis, based on the trauma and injury severity score (TRISS), was nearly identical in both groups: 98.5% for the intubated patients and 98.4% for those without intubation. The similarity of the general patient characteristics in the two groups supports the conclusion that they were comparable and nearly identical.

**Table 1:** The demographic and clinical data for the injured patients with or without intubation before hospitalization (600 patients per group)

	Not intubated	Intubated	Total	*P *value
Patients (no.)	600	600	1,200	
Age in years (MV, SD)	39.5 ± 17.3	38.6 ± 16.9	38.8 ± 17.1	0.69
Male (%)	79	79	79	1.00
ISS (MV, SD)	15.1 ± 4.9	15.1 ± 5.0	15.05 ± 5.0	0.77
Blunt trauma (%)	95.8	93.5	94.7	0.72
AIS head 1 to 3 (%)	20.2	20.2	20.2	1.00
AIS face 1 to 3 (%)	1.3	4.3	2.8	0.21
AIS thorax 1 to3 (%)	46.8	46.8	46.8	1.00
AIS abdomen 1 to 3 (%)	7.5	7.5	7.5	1.00
AIS extremities, including pelvis 1 to 3 (%)	51.2	51.2	51.2	1.00
AIS soft tissue 1 to 3 (%)	0.3	0.3	0.3	1.00
Prehospital respiratory rate (MV, SD)	16.6 ± 4.3	16.5 ± 5.1	16.6 ± 4.7	0.13
Prehospital blood pressure, mm Hg (MV, SD)	128.8 ± 23.2	124.5 ± 26.9	126.7 ± 25.2	≤ 0.009
Prehospital heart rate (MV, SD)	91.1 ± 16.7	96 ± 20.2	93.5 ± 18.7	≤ 0.001
TRISS survival prognosis (%)	98.6	98.5	98.6	0.41

Values shown as mean **± **standard deviation or percentage of the group. AIS, abbreviated injury scale; ISS, injury severity score; MV, mean value; SD, standard deviation; TRISS, trauma and injury severity score.

### Prehospital and emergency department treatment

The blood pressure and heart rate at hospital admission were significantly better in the group without intubation (Table 2). Volume replacement (crystalloid and colloidal) was significantly higher in the group with intubation (not intubated, 911.3 ± 633.3; intubated, 1,573.8 ± 868.4; *P *≤ 0.001). The hemoglobin level, prothrombin ratio, and platelet count were significantly decreased in the group of patients with prehospital intubation (Table 2). The body temperatures of the patients measured at the emergency department were lower in the group of intubated patients (not intubated, 36.7 ± 0.7; intubated, 36.1 ± 0.9; *P *≤ 0.001).

**Table 2:** Group-specific patient data from treatment at the accident site, in the emergency department, and during initial surgical treatment

	Not intubated	Intubated	Total	*P *value
Prehospital volume received (%)	91	94.8	92.9	0.01
Prehospital volume (ml, MV, SD)	911.3 ± 633.3	1,573.8 ± 868.4	1,242.6 ± 828.8	≤ 0.001
Prehospital rescue time (minutes, MV, SD)	64.8 ± 41.5	82.3 ± 41	73.3 ± 42.2	≤ 0.001
Blood pressure (mm Hg) in the emergency trauma room (MV, SD)	135.4 ± 22.7	123.6 ± 22.4	129.5 ± 23.3	≤ 0.001
Heart rate at admission to hospital (MV, SD)	90.4 ± 16.3	84.5 ± 16.8	87.4 ± 16.8	≤ 0.001
Hb at admission to hospital (mg/dl, MV, SD)	13.4 ± 2.1	12.2 ± 2	12.8 ± 2.1	≤ 0.001
Prothrombin ratio (%; MV, SD)	92.3 ± 16.1	85.7 ± 16.6	89 ± 16.7	≤ 0.001
Base excess (MV, SD)	-1.2 ± 2.9	-2.1 ± 3.7	-1.8 ± 3.4	≤ 0.001
Platelet count/nl at admission to hospital (MV, SD)	234,094.6 ± 74,398.3	201,908.6 ± 67,408.2	217,895.4 ± 72,737.1	≤ 0.001
Lactate at admission to hospital (MV, SD)	2.6 ± 3.2	2.8 ± 4	2.8 ± 3.7	0.01
Temperature at admission to hospital (°C, MV, SD)	36.7 ± 0.7	36.1 ± 0.9	36.4 ± 0.9	≤ 0.001
Prehospital use of catecholamines (%)	0	5.3	2.7	≤ 0.001
Prehospital chest tube (%)	0.7	7.3	4	≤ 0.001
Prehospital cardiopulmonary resuscitation (%)	0	0.8	0.4	0.03
Prehospital intravenous sedation (%)	71.7	97	84.3	≤ 0.001

Values shown as mean **± **standard deviation or percentage of the group. Hb, hemoglobin; ml, milliliter; MV, mean value; SD, standard deviation.

Prehospital measures, such as chest tubes, sedation, and cardiopulmonary resuscitation, were significantly higher in the group with intubation (Table 2). Prehospital administration of catecholamines was significantly higher in the group of intubated patients (not intubated, 0; intubated, 5.3%; *P *≤ 0.001). The rescue time (defined as the time until arrival at the emergency department) was significantly higher for the intubated group than for those without intubation (not intubated, 64.8 ± 41.5 minutes; intubated, 82.3 ± 41 minutes; *P *≤ 0.001) (Table 2).

### Intensive care and outcome

Almost the same number of patients were treated with operative therapy in both groups (Table 3), and the same applies to the number of emergency surgeries. In both cases, no significant difference could be detected. The groups differed significantly in their further clinical courses, as assessed by length of stay in the intensive care unit, days intubated in the intensive care unit, and ventilator-free days in the first 30 days after trauma. All of these measures indicated a less-desirable clinical course in the prehospital-intubation group. It should be mentioned that the days intubated for the patients without prehospital intubation consisted mainly of intubation during surgery and postoperative ventilation.

**Table 3:** The intensive-care outcomes of the trauma patients with or without prehospital intubation

	Not intubated	Intubated	Total	*P *value
Emergency surgery (%)	0.8	0.3	0.6	0.3
Surgery (%)	82.8	86	84.4	0.13
Stay in intensive care unit (%)	87.2	95.2	91.2	≤ 0.001
Days in intensive care unit (MV, SD)	4.1 ± 5.9	5.9 ± 7.1	5 ± 6.6	≤ 0.001
Days intubated (MV, SD)	1.3 ± 4.9	3 ± 5.6	2.2 ± 5.4	≤ 0.001
Ventilator-free days (30 days, MV, SD)	28.6 ± 4.6	26.9 ± 5.4	27.8 ± 5.1	≤ 0.001
Organ failure (%)	9.1	23.4	16.2	0.001
Multiple organ failure (%)	4.3	9.8	7	≤ 0.001
Sepsis (%)	1.5	3.7	2.6	0.02
Died in hospital (%)	1	0.5	0.8	0.32
Days in hospital (MV, SD)	20 ± 15.1	22.4 ± 15.1	21.2 ± 15.5	≤ 0.001
Costs (€; MV, SD, referring to days of stay)	15,911.1 ± 11.1	20,061.4 ± 17,493.3	18,060 ± 14,905.5	≤ 0.001

Values shown as mean **± **standard deviation or percentage of the group. MV, mean value; SD, standard deviation.

Process parameters in intensive care units, such as sepsis (not intubated, 1.5%; intubated, 3.7%; *P *≤ 0.02), multiorgan failure (not intubated, 4.3%; intubated, 9.8%; *P *≤ 0.001) and especially single-organ failure (not intubated, 9.1%; intubated, 23.4%; *P *≤ 0.001), all indicated significantly less-desirable outcomes in the intubated patients. However, mortality did not show any significant differences (not intubated, 1%; intubated, 0.5%; *P *= 0.32). The total length of stay in the hospital (in days) was significantly higher for the intubated group than for those without intubation. Additionally, the total hospitalization expenses were significantly higher in the intubated patients (not intubated, €15,911.1 ± 11,141; intubated, €20,061 ± 17,493.3; *P *≤ 0.001).

## Discussion

The aim of this study was to examine the differences in organ failure, sepsis, ICU stay, and mortality attributable to prehospital intubation. The study was limited to patients for whom it was possible to show an advantage or disadvantage of intubation, as assessed by reduced severity of injury. A matched-pairs analysis was performed to compare two groups that had the same initial conditions but received different therapy, thus assessing the effects of the different airway-management approaches.

The aim of prehospital intubation after trauma is to supply the organs with sufficient oxygen to prevent early tissue hypoxia and the resulting mortality and morbidity [13]. The clear recommendations for prehospital intubation discussed in the literature (for example, in cases of severe traumatic brain injury or severe thoracic trauma) refer to severely injured patients [14,15]. A frequent concern of emergency-response teams is the unpredictable and unknown total severity of injuries, which can result in deleterious undertreatment, particularly of patients with severe traumatic brain injuries. In this respect, Sise [16] concluded that prehospital intubation is rarely associated with risks in trauma patients. Based on the available data, we could not determine whether the injury patterns that were obvious at the accident site or the distance to the nearest trauma center influenced the decision to intubate. It should be noted that the injuries to the individual body regions were exactly the same for both groups, except for the insignificant frequency of facial injuries in the intubated group.

The present study shows that prehospital intubation can be a risk factor in the treatment of a patient after trauma. Of course, it should be remembered that the prehospital decision to intubate must often be made on a case-by-case basis at the accident site. However, the present study shows that in matched groups of trauma patients, intubation was associated with a number of complications. The rescue time for intubated patients increased significantly. In this context, it could be argued that the longer rescue time caused by prehospital intubation was compensated by the time saved in the hospital; that is, by not needing to intubate for emergency surgery. This argument is not relevant for the patients described here, however, because most of these moderately injured patients did not require in-hospital intubation. This conclusion is bolstered by the small numbers of patients in both groups who needed emergency surgery. The amount of prehospital volume replacement also increased significantly; consequently, the coagulation status of the patients decreased significantly. The connection between increased volume replacement and the resulting coagulation impairment was described by Dutton *et al*. [17,18]. In this context, the findings of Brohi *et al*. [19] must be considered: in a retrospective study, they found that the trauma itself could cause acute coagulopathy, independent of the volume applied.

The present data show that no hemorrhagic shock was present at the initial arrival of the emergency-response team at the accident site. Therefore, it is most likely that no direct reason existed for extensive volume replacement. The assumption that induced anesthesia can lead to circulatory insufficiency is supported by the finding that the intubated patients received significantly more catecholamines than did the not-intubated patients, who did not receive catecholamines. The same conclusion applies to the administration of sedative medication. In his review, Mort [7] mentioned possible circulatory insufficiency after anesthesia, but he was not referring to mandatory intubation after trauma in that context. In addition, the data show that the intubated patients arrived at the hospital with significantly higher rates of hypothermia. This complication can also contribute to impaired coagulation.

It is noteworthy that the intubated patients required cardiopulmonary resuscitation significantly more often than did the not-intubated patients. Mort [20] also addressed this connection, but it was not specifically related to trauma patients at accident sites in his study. In cases of cardiopulmonary resuscitation, it is important to remember that a patient's prehospital condition can always decline, thus requiring new exit criteria for the emergency team. The injury severity was the same in both of the studied groups. The need for emergency surgery or surgery during the course of treatment, an indirect marker of injury severity, was not significantly different between the groups.

A striking finding of this study was that the prehospital-intubation patients had significantly more multiorgan failure, sepsis, and (especially) single-organ failure than did the similarly injured patients who were not intubated. The complication rate for the intubated patients may have played a role. The incidence of pneumonia in the intubated patients is particularly significant. Aside from the clinical situation as a whole, this pneumonia may have been responsible for the increased incidence of sepsis and multiorgan failure; however, because the data came from a registry-based, retrospective analysis, this connection remains speculative. In the current literature, Carr *et al*. [21] described the intubation rate after trauma and the resulting pneumonia as a single risk factor. They found an increased pneumonia risk of 20% per hour in intubated patients after blunt trauma, compared with a control group with the same injury severity and with or without a shorter intubation time.

The elevated complication rate among the prehospital intubation patients was also reflected by longer stays in the ICU, more days of intubation, and longer hospital stays. Conversely, the number of ventilator-free days during the first 30 days after trauma was higher among those without intubation. Unfortunately, this connection cannot be examined by using the current literature, because of the lack of data on moderately injured trauma patients. Hospitals may compensate for the worsened conditions created by prehospital intubation. No significant difference in mortality was found between the two groups. The increased clinical expenditures were reflected by the significantly higher hospitalization costs (approximately €4,000 per patient) in the intubated patients.

It also is striking that in the present study, the patients who were intubated by the emergency-response team at the accident site received significantly more therapy, such as chest tubes. In such cases, the emergency-response team's concern about causing a tension pneumothorax in the intubated patients after intubation may have been an issue. The restricted on-site diagnostic capabilities support such concerns. Unfortunately, the current literature on this issue is also scarce. Because the incidence of thoracic injuries was the same in both groups, thoracic injury alone could not have explained the differences in therapeutic decision making.

Finally, intubation is certainly not the treatment of choice for patient pain control. Analgesics that are applicable in emergency medicine are preferable.

According to the basic ATLS guideline to "do no further harm," the present data support reducing the intubation rate in mildly injured trauma patients [3].

There are several limitations to our retrospective study:

1. A TRISS calculation could be performed only in 46% of the participating trauma centers. This is because, among other issues, the respiratory rate was documented on-site by the physicians in only 60% of the cases. However, because of the present study's selection criteria, a survival probability less than the defined 98% was not to be expected, because only moderately injured patients were involved.

2. Regarding the coagulation analysis, the DGU Trauma Registry documents only the prothrombin ratio; therefore, it is the only coagulation information available for analysis. Other laboratory values that might be of interest with respect to coagulation (for example, fibrinogen and protein C) are not documented at all in the Trauma Registry.

3. All the patients were treated by physicians at the accident sites. However, it remains unclear which specialty (for example, trauma surgeon or anesthetist) the accident-site physician represented and his or her educational background. In Scandinavian countries, for example, only anesthetists are allowed to work as physicians at an accident site. In contrast, in German-speaking countries, any physician (for example, a surgeon or anesthetist) with an additional certificate in emergency medicine is authorized to work as an emergency doctor at an accident site. In Germany, no emergency-medicine specialty exists, as it does in Anglo-American countries, for example. Based on the data from the DGU trauma registry, it cannot be determined whether emergency physicians trained and qualified in anesthesia achieve better patient outcomes because they frequently use intubation in clinical situations. In Germany, more anesthetists than surgeons are involved in accident treatment.

4. Individual therapy decisions (for example, the decision to intubate) at the accident site could not be reconstructed because of the anonymous nature of the data, nor could the distances to a hospital be determined retrospectively.

5. A subgroup analysis to address whether there was an increased occurrence of pneumonia in patients after thoracic trauma could not be conducted because related values (for example, pneumonia yes/no) are not collected in the DGU trauma registry. The same applies to preexisting illnesses and whether they had an influence on patients' clinical courses (for example, cardiopulmonary resuscitation or changes in the base excess).

Finally, we conducted a retrospective analysis; associations, but not causalities, can be ascribed to the given data. Future prospective, randomized studies are needed to clarify the advantages or disadvantages of intubation therapy in injured patients at the accident site.

## Conclusions

Prehospital intubation after trauma is likely an additional risk factor. Patients with a sufficient specific oxygen-uptake rate seem to benefit from rapid transport to a trauma center. Therefore, the out-of-hospital therapy should be limited to the stabilization of vital parameters. Intubation does not lead to better outcomes in trauma patients who do not have clear indications for intubation.

## Key messages

• Prehospital intubation in trauma patients is associated with a number of risks and should be critically weighed, except in cases in which absolute indicators exist, such as posttraumatic apnea.

• When applied uncritically, prehospital intubation in trauma patients can have a negative effect on the clinical course (for example, elevated sepsis rate, higher rate of organ failure and multiple-organ failure, longer stay at the ICU, and altogether longer in-hospital stay).

• Because of the extended rescue time, an uncritically applied prehospital intubation in the trauma patient leads to delayed in-hospital patient care.

• Prehospital intubation in trauma patients leads to higher expenses in patient care.

• Because of prehospital intubation in trauma patients and, for example, the associated higher volume replacement, the coagulation system is negatively affected, and therefore, the starting conditions in the hospital are deteriorated.

## Abbreviations

ACCP-SCCM: American College of Chest Physicians/Society of Critical Care Medicine; AIS: abbreviated injury scale; ATLS: advanced trauma life support; DGU: German Association for Trauma Surgery: EAST: Eastern Association for the Surgery of Trauma; ED: emergency department; GCS: Glasgow Coma Scale; Hb: hemoglobin; ICU: intensive care unit; ISS: injury severity score; MOF: multiple organ failure; MV: mean value; SaO_2_: arterial oxygen saturation; SD: standard deviation; SOFA: sequential organ-failure assessment; SPSS: statistical package for the social sciences; SURG: surgery; TRISS: trauma and injury severity score.

## Competing interests

The authors declare that they have no competing interests.

## Authors' contributions

BH conceived the study, designed the trial, obtained research funding, provided statistical advice on study design, analyzed the data, and drafted the manuscript. CW, AW, and MDK conceived the study, designed the trial, and obtained research funding. SL conceived the study, designed the trial, and obtained research funding, supervised the conduct of the trial and data collection, provided statistical advice on study design, and analyzed the data. SR supervised the conduct of the trial and data collection. RL provided statistical advice on the study design and analyzed the data, and supervised the conduct of the trial and data collection. BH takes responsibility for the article as a whole. All authors contributed substantially to manuscript revision. All authors have read and approved the final manuscript for publication.
